# The Profile of Bacterial Infections in a Burn Unit during and after the COVID-19 Pandemic Period

**DOI:** 10.3390/antibiotics13090823

**Published:** 2024-08-30

**Authors:** Corina Musuroi, Silvia-Ioana Musuroi, Luminita Baditoiu, Zorin Crainiceanu, Delia Muntean, Adela Voinescu, Oana Izmendi, Alexandra Sirmon, Monica Licker

**Affiliations:** 1 Multidisciplinary Research Center of Antimicrobial Resistance, Microbiology Department, “Victor Babes” University of Medicine and Pharmacy, 300041 Timisoara, Romania; corina.musuroi@umft.ro (C.M.); muntean.delia@umft.ro (D.M.); adela.voinescu@umft.ro (A.V.); licker.monica@umft.ro (M.L.); 2 Microbiology Laboratory, “Pius Brinzeu” County Clinical Emergency Hospital, 300723 Timisoara, Romania; 3 Doctoral School, “Victor Babeș” University of Medicine and Pharmacy, 300041 Timisoara, Romania; 4 Epidemiology Department, “Victor Babes” University of Medicine and Pharmacy, 300041 Timisoara, Romania; baditoiu.luminita@umft.ro (L.B.); alexandra.sirmon@gmail.com (A.S.); 5 Department of Plastic Surgery, “Victor Babes” University of Medicine and Pharmacy, 300041 Timisoara, Romania; crainiceanu.zorin@umft.ro; 6 Epidemiology Department, “Pius Brinzeu” County Clinical Emergency Hospital, 300723 Timisoara, Romania

**Keywords:** burn, antimicrobial resistance, non-fermenting Gram-negative bacteria, pandemic period

## Abstract

Infections represent a major complication for burn-injured patients. The aim of this study was to highlight the changes in the incidence and antimicrobial resistance of bacterial strains isolated from burn patients, at the end of the COVID-19 pandemic, in relation to the antibiotics used during the pandemic. A comparative analysis of the demographic data and the microorganisms identified in the clinical samples of two groups of burn patients admitted to a university hospital in Romania was carried out. The first group consisted of 48 patients and the second of 69 patients, hospitalized in January–August 2020 and 2023, respectively. The bacterial species with the highest incidence were *S. aureus*, *A. baumannii*, *Pseudomonas* spp. The significant changes between 2023 and 2020 are reflected in the increase in the frequency of non-fermentative Gram-negative bacteria, especially *S. maltophilia*, and the increase in antimicrobial resistance of *Pseudomonas* and *Klebsiella* spp. *Klebsiella* spp. did not change in frequency (7%), but there was a significant increase in the incidence of *K. pneumoniae* strains with pan-drug resistant behaviour to antibiotics (40%), including colistin. The phenomenon can be explained by the selection of specimens carrying multiple resistance genes, as a result of antibiotic treatment during the COVID-19 period. The post-pandemic antimicrobial resistance detected in burn patients indicates the need for permanent surveillance of the resistance trends, primarily due to the limited therapeutic options available for these patients.

## 1. Introduction

Infection is a major complication in burn injuries, and it is estimated that up to 75% of deaths that occur after burns are related to infection [1].

Although exposed burned tissue is susceptible to contamination with microorganisms from the gastrointestinal and upper respiratory tracts, Vindenes [2] indicates gradual changes in the colonization of burn wounds, whose progression must be followed in dynamics. While staphylococci and α-haemolytic streptococci are prevalent upon hospital admission and throughout the first week of hospitalization, these bacteria are gradually replaced in the following weeks by species of *enterococci*, pathogenic opportunistic Gram-negative bacteria (GNB), particularly *P. aeruginosa*, *Acinetobacter* spp. and *E. coli*, and also *Candida* spp. [2].

The transition from colonization to infection depends mainly on three factors: the bacterial colonization level, the pathogen virulence, and the burned patient’s immune defence level [3]. In fact, the burn wound is a complex microenvironment consisting of necrotic tissue and plasma-derived exudate, components that create a niche environment in which pathogens with high metabolic versatility can successfully proliferate [4].

It is known that opportunistic pathogens, such as *Pseudomonas aeruginosa*, *Acinetobacter baumannii*, and *Staphylococcus aureus*, are involved in the colonization of burn wounds. Moreover, Gonzalez et al. [4] show that *P. aeruginosa* is remarkably efficient at proliferating in the exudate of burn wounds, adapting to the environment through the activation of the catabolism of lipids and collagen, a fact that could not be proved for other tested pathogens.

A meta-analysis of published data on chronic wounds reveals that nearly 80% are associated with biofilm formation [5]. Most studies associate chronic burn wounds with biofilm formation in the stage of persistent inflammation, through the accumulation of immune cells unable to destroy them [6,7]. Biofilms have also been detected in acute wounds, but the recorded incidence was low (6%), some in vivo studies indicating that biofilms can form in acute wound models within the first 3 days post-trauma [8,9]. Likewise, in vivo models have shown that biofilm formation in burn wounds can precede systemic infection [10,11]. This suggests that biofilms may play a role in the progression of acute infection, acting as a launch area for deeper tissue invasion associated with bacteraemia and sepsis [10,12,13].

On the other hand, respiratory tract burns are listed among the most serious injuries to the human body, oftentimes accompanying severe skin burns, increasing morbidity and mortality, and septic shock can be the cause of death in most of these patients [14,15].

The COVID-19 pandemic has presented a public health challenge to which countries have responded by implementing different measures to handle the crisis [16]. Care protocols for burned patients testing positive for COVID-19 have varied according to the public health model used in each country [17]. A study on 234 burn centres from 43 countries indicated that, while the COVID-19 pandemic produced a change in surgical priorities, it did not target burn care units, which were maintained in constant functionality [16]. It is worth noting, however, that the appointments of surgical interventions were affected, with potential negative impact on the management of the burn patient and consequences on the evolution of the disease.

Another study, conducted on a group of 1472 burn patients, of which half had an associated COVID-19 infection, indicated that burned patients with COVID-19 did not show a significant increase in mortality compared to those testing negative. However, it has been observed that co-infection with this virus was associated with an increase in the incidence of infections, thrombosis, and hypertrophic scars in burned patients [18].

Although 2020 was dominated by the SARS-CoV-2 infection, which represented a premise for the reduction in antibiotic consumption, 2020 was a missed opportunity to reduce the antibiotic consumption in Romania, as a preferential use of broad-spectrum antibiotics (penicillins with inhibitors, large-spectrum cephalosporins, carbapenems) and even reserve antibiotics (like colistin or vancomycin) was noticed. All of these posed a major risk for the selection of multi-drug-resistant (MDR) pathogens. When comparing pre-pandemic (2017) with post-pandemic (2021) EARS-Net data [19], an increasing trend of antimicrobial resistance (AMR) was noticed, especially for *K. pneumoniae* and *A. baumannii*, not only in Romania, but also in other southern and eastern parts of the European Region. At the same time, the percentage of AMR is high for methicillin-resistant *S. aureus* (MRSA) and vancomycin-resistant *Enterococcus* (VRE), but the trend is stationary.

In this context, the present study investigated the differences in the aetiology, the types of infections, and the AMR, in relation to the antibiotics used during the pandemic, and the variables predicting the negative evolution of patients hospitalized in the Burn Functional Unit (BFU), during and after the COVID-19 pandemic. The aim of this study also lay in establishing the correlations between the characteristics of the species identified in the clinical samples and the location of the infections, considering the AMR profile, the biofilm-forming potential, and the consequences on the treatment and evolution of these patients.

## 2. Results

This study aimed at the comparative description of the patients with infections and the microorganisms identified in the samples of two groups of burn patients hospitalized in the BFU of the “Pius Brinzeu” Emergency County Clinical Hospital Timișoara (SCJUPBT), in relation to the antibiotics used during the pandemic. Group 2020 (G20) consisted of 48 patients admitted between 1 January and 31 August 2020, while Group 2023 (G23) included 69 patients admitted between 1 January and 31 August 2023.

A total of 63 burn-injured patients were admitted to BFU between January and August 2020, of which G20 patients, i.e., those diagnosed with infections, accounted for 76.19% of the total. Comparably, 92 burn-injured patients were admitted between January and August 2023, of which G23 represented 75%.

The demographic and prognostic data of the patients on admission are presented in Table 1.

It can be observed that, with a number at least double that of women, the prevalence of male patients is significant and applicable to both study groups. 

As regards age on admission, the median age associated with G23 was significantly lower than that of G20. This behaviour was determined by two factors: the significant increase in the number of young patients, aged 20–29, and the significant decrease in the number of patients aged 80–89 belonging to G23.

The median length of stay was comparable for the two groups, with a total number of hospitalization days for G23/G20 patients of 2078 and 1535 days, respectively.

No significant differences were recorded between G23 and G20 regarding the total body surface area burned. It must be noted, however, that the median fatality, calculated according to the ABSI (Abbreviated Burn Severity Index) score on admission, was lower in 2023 relative to 2020. This could be explained by an increase in the number of hospitalized patients in the fatality rate of 0–2%, with a low risk of fatal outcome, belonging to G23 (*p* = 0.049). The distribution by fatality categories is shown in Table 2.

Likewise, direct, average, highly statistically significant correlations were established between Body Surface and Fatality Category (rho = 0.536, *p* < 0.001), as well as between Age and Fatality Category (rho = 0.445, *p* < 0.001).

As regards discharge status, no statistically significant differences were recorded between G23 and G20. The data indicated a similar incidence among patients discharged with cured status (47.83%/43.75%, *p* = 0.709), the improved/stationary evolution (30.44%/18.75%, *p* = 0.052), and the deceased (21.74%/37.50%, *p* = 0.094).

In a logistic regression model explaining 80% of the data variation (*p* = 0.684) (Hosmer and Lemeshow test), the independent predictive variables for negative evolution were age, burned body surface, mechanical ventilation, haemodialysis, and the infection with *Acinetobacter* spp. (Table 3). By contrast, wound dressing ≥10% of the body surface (BS) met the statistical criteria for protection factor.

### 2.1. Clinical Samples

As concerns clinical samples, no significant statistical differences were recorded regarding the frequency rates of the main types of specimens collected for the two groups of patients (G23/G20), namely wound secretions (*p* = 0.457), blood cultures (*p* = 0.274), and bronchial aspirates (*p* = 0.321).

By contrast, a significant increase in the percentage of positive urine cultures applicable to G23—8.30% [95%CI, 5.21–12.41%]—was noticed, relative to 3.56% [95%CI, 1.55–6.89%] (*p* = 0.034), recorded for G20.

Also, the share of blood cultures among the samples collected from G23 patients decreased significantly compared to G20, strictly for patients with I/II-degree burns: 7.69% versus 29.63% (*p* = 0.039). 

### 2.2. Clinical Isolates

The number of isolates identified in the clinical samples was 245 for G20, respectively, 273 for G23.

**Figure 1:**
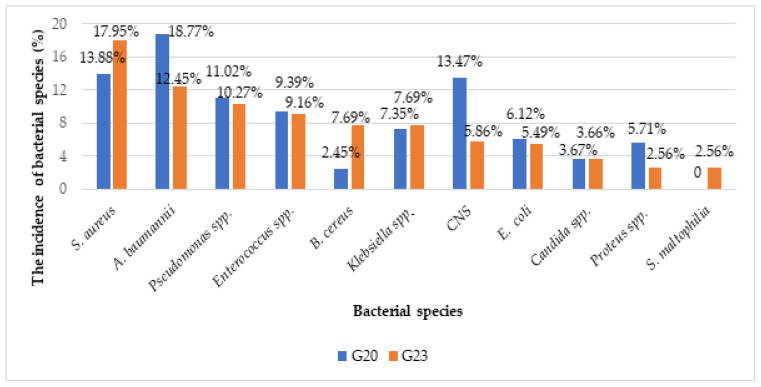
Distribution of clinical isolates identified in G23 versus G20 (%); CNS: coagulase negative staphylococci.

As concerns the comparative incidence of bacterial species identified in G23 versus G20, the first five species were *S. aureus* (17.95%/13.88%, *p* = 0.231), *A. baumannii* (12.45/18.77%, *p* = 0.052), *Pseudomonas* spp. (10.27%/11.02%, *p* = 0.888), *Enterococcus* spp. (9.16%/9.39%, *p* = 1.00), and *Klebsiella* spp. (7.69%/7.35%, *p* = 0.869), without statistically significant differences between the two groups (Figure 1).

Significant differences were, however, recorded for species with a lower incidence. Respectively, the incidence of *B. cereus* increased from 2.45% [95%CI, 0.90–5.25%] in G20, to 7.69% [95%CI, 4.82–11.52%] in G23 (*p* = 0.009), considering the more frequent isolation in wound secretions. An opposite direction was recorded for coagulase negative staphylococci (CNS) strains which decreased significantly to 5.86% [95%CI, 3.39–9.34%] in G23, relative to 13.47% [95%CI, 9.46–18.39%] in G20 (*p* = 0.004), by the decrease in isolation in wound secretions (Table 4).

Also, the emergence of *S. maltophilia* strains marked an incidence of 2.56% [95%CI, 1.04–5.21%] for G23, relative to the absence of this species in G20 (*p* = 0.016), explained by the increase in isolation in the bronchial aspirates of G23 patients (*p* = 0.043) (Table 4). 

It was indicated that changes in the incidence of G23 isolated species relative to G20 were mostly due to the infections occurring in patients with third- and fourth-degree burns. For them, there was a significant increase in the incidence of *B. cereus* (*p* = 0.021) and *S. maltophilia* (*p* = 0.015), in addition to a decrease in CNS strains (*p* = 0.024).

The bacterial infection profile varied according to the type of isolates. Table 4 shows the frequency, in descending order, of the main genera/species from G23, respectively G20, in the main clinical samples, as well as the statistical significance of the comparison.

GNB strains outnumbered those of *Gram-positive cocci* (GPC) for both G23 (51.28%/45.05%) and G20 (54.69%/41.22%). Fungi remained at low levels (3.66%/4.08%), without statistically significant differences (see Table 5).

The non-fermenting GNB species represented approximately 1/3 of the total number of strains in G23 and G20 (27.84%/30.20%), with the presence of *Acinetobacter*, *Pseudomonas S. maltophilia* (G23, G20), *Sphingomonas paucimobilis* (G23) and *Burkholderia caepacia* (G20).

The presence of non-fermenting GNB was significant in wound secretions, where *Acinetobacter* and *Pseudomonas* accounted for approximately 1/4 of the total G23/G20 strains (23.81/26.96%), as well as in bronchial aspirates with an incidence of 53.33% in G23 and 47.05% in G20. The emergence of two new species in bronchial aspirates in G23, i.e., *Stenotrophomonas maltophilia* and *Sphingomonas paucimobilis*, was also recorded (Table 4).

As regards resistance phenotypes, no significant variations between G23 versus G20 were recorded for *A. baumannii*, *Staphylococcus aureus*, *CNS*, and *Enterococcus* spp. (Table 6).

But specific changes in resistance behaviour were recorded for *Pseudomonas* spp. and *Klebsiella* spp. A significant increase in multi-drug-resistant (MDR) *Pseudomonas* strains was reported—44.90% for G23, relative to 18.52% for G20 (*p* = 0.047). On the other hand, *Klebsiella* species experienced a significant increase in the number of pan-drug-resistant (PDR) strains for G23 (40.91%), relative to G20 (5.55%, *p* = 0.013).

## 3. Discussion

Thermal injuries comprise a pathology that affects over 8 million people worldwide, with 265,000 deaths annually attributed to burns caused by fires [20]. Injuries associated with burn wounds are caused by the attack of physical agents on the human body, accidentally and rarely intentionally exposed to these agents.

The pandemic period has been associated with an increased use of antibiotics, overcrowded hospitals, softening of the surveillance and infection control measures (other than COVID-19), changes in patients’ behaviour, and disruptions in drug supply chains, which ultimately led (contrary to expectations) to an increasing infection rate caused by antibiotic-resistant bacteria [19,21].

Despite adopting several measures to counteract the resistance evolution, AMR still poses a major public health problem in the post-pandemic period [22].

The comparison drawn between the two groups of patients evaluated in this study highlights major age-related differences. The number of patients of working age (up to 59 years) grew significantly in the case of G23 relative to G20 (over 70.00% compared to 50%), particularly given the increase in those aged under 30 (from 2.08% to 15.94%). This could be explained by the increased availability of the population to access the health care system, as the risk of the COVID-19 infection decreased. This availability also explains the increased rate of patients included in the 0–1%/2% fatality category in 2023. Men outnumbered women in both study groups, particularly due to the type of activity incurring higher risks of exposure to flame, but also because of certain addictions (alcohol consumption, drugs) which increase the risks of accidents. This behaviour was a characteristic of the pandemic period, considering the more severe forms of COVID-19 experienced by men compared to women, as indicated by literature data [23] which corroborate that the percentage of hospitalized men could be higher and that men died more often.

The percentage of patients discharged with positive evolution (cured/improved) was comparable between G23 and G20 (*p* = 0.061), with up to approximately 50% of the cases discharged under 30 days of hospitalization. This indicates that the management of burn-injured patients was successfully adapted to the challenges posed by the pandemic. It was observed that in both study groups approximately 60% of those who died were hospitalized for less than 30 days, and of those 2/3 for less than 10 days. This underlines that the fatal outcome was determined by the acute injury and the parameters of the patient on admission and not due to developmental complications or treatment.

The problem concerning burn-injured patients is related to the ratio between the hospitalization period and the discharge status, which is optimal when good health and functionality are obtained in as short a period of hospitalization as possible. This ratio is affected, however, by a range of independent variables that intervene from the beginning of the disease and determine the evolution of the patient, such as burn degree, affected body surface, associated diseases, and pathology-derived complications, including those of an infectious nature.

The independent predictive variables for negative evolution identified in this study were age, burned body surface, mechanical ventilation, haemodialysis, and infection with *Acinetobacter* spp. No predictions about the time of evolution were made.

The analysis of the set of positive clinical samples indicated, as expected, that the main types of samples consisted of wound secretions (G23—60.07%). Positive blood cultures, bronchial aspirates, and urine cultures were also recorded, with much lower incidence, up to approximately 20%, signifying the occurrence of infectious complications (systemic, respiratory tract and urinary tract infections) in these patients.

The highest incidence among bacterial species was shown by *S. aureus*, *A. baumannii*, and *Pseudomonas* spp. (10–20%), corroborating that the species distribution followed the pattern developed in previous studies conducted by our team [24,25]. While *S. aureus*, *Enterococcus* spp. and *P. aeruginosa* were identified as prevalent species in wound secretions, *A. baumannii*, *Pseudomonas* spp. and *S. maltophilia* (G23) prevailed in bronchial aspirates. Lower frequencies (<10%) were recorded for *Klebsiella* spp., *E. coli*, and *Proteus* spp., in all types of clinical samples.

A regular presence of *Candida* species was also observed (≈3.5%), but their incidence in wounds was low (G23—1.19%). The explanation possibly lies in the presence of a combination of factors in the burned tissue, including those of a microbial nature, which exert a negative impact on the proliferation of *Candida* spp. [10]. Literature data suggest that the incidence of *P. aeruginosa* in burn injuries can prevent the proliferation of *Candida* spp., with biofilms produced by *P. aeruginosa* potentially colonizing fungal hyphae and leading to their death [26,27]. Nazik et al. also have experimentally shown that bacteriophages can exert an inhibitory effect on the formation of fungal biofilms, using a *P. aeruginosa* strain that contained a phage with a destructive action on *C. albicans* [28].

This study highlighted a significant incidence of *S. maltophilia* strains in G23, preva-lent in bronchial aspirates (13.33%/0%, *p* = 0.043) and more rarely detected in wound secretions (1.79%/0%).

*S. maltophilia* is a bacterium with multiple innate resistance mechanisms, which often proliferates in hospital environments. Its increased frequency is likely a result of the selective pressure caused by the overuse of broad-spectrum (cephalosporins, carbapenems) or even reserve antibiotics (such as colistin), particularly in critical care settings. Furthermore, the COVID-19 pandemic increased the need for invasive medical equipment like ventilators and caused longer hospital stays, particularly in intensive care units. *S. maltophilia* is a well-known opportunistic pathogen, which frequently causes catheter-related infections and pneumonia linked to the use of ventilators in hospital settings [12,29]. Under these circumstances, the excessive use of antibiotics probably favoured the increased development of *S. maltophilia*.

The presence of *S. maltophilia* in the clinical samples of burn-injured patients is a cause for concern, especially knowing that the World Health Organization (WHO) has listed *S. maltophilia* among the significant emerging pathogens for public health [30,31]. This species ultimately impairs the efficiency of most antibiotics [32]. Pulmonary damage and burn injury infection of immunocompromised patients, present in this study, were also reported in literature data [33,34], being associated with mortality rates of up to 69% in systemic infections [12,29].

A significant increase in *B. cereus* was recorded in the G23 wound secretion, relative to G20 (*p* = 0.010). *B. cereus* is a well-known, commonly found environmental pathogen, which can be responsible for unintentional wounds contamination by means of contaminated objects, equipment, or surroundings in burn units. Furthermore, *B. cereus* can be involved in biofilm development, especially on wound surfaces or medical equipment, being responsible for more persistent infections. The increasing emergence of *B. cereus* strains in 2023 could be caused by multiple factors, such as cross contamination between patients, contaminated gloves of medical nurses, as suggested by another study [35], or even the more accurate identification of the microorganism with the purchase of the MALDI TOF equipment in our case. At any rate, *B. cereus* isolates did not show acquired resistance mechanisms to antibiotics in our case and they did not pose treatment problems.

Nevertheless, there are studies showing the evolutionary potential of *B. cereus* towards an MDR behaviour in burn-injured patients. It has been indicated that MDR species increase the death rates of patients with burn-related sepsis from 42% to 86% [35].

While AMR behaviour has not changed fundamentally, there are specific deviations associated with increased resistance, which are important for the incidences and phenotypes described.

It must be noted, however, that this phenomenon has not exclusively affected the patients admitted to our hospital. This observation is also widely referenced in literature, which mentions that the widespread use of broad-spectrum antibiotics during the COVID-19 pandemic did not bring explicit clinical benefits, but fostered bacterial and fungal superinfections, and AMR development, especially to GNB [17,36,37]. In the post-pandemic period, the bacterial resistance to antibiotics has developed [22,38] affected by a range of factors including changes in medical practices, the revival of healthcare systems, and the adjustment of public health policies. The CARMIAAM report [39] indicates that pandemic-stricken Romania recorded, for the first time in 2021, the highest consumption of antibiotics among EU/EEA states. The first three groups of antibiotics were penicillins, macrolides, and cephalosporins. The improper or excessive use of large-spectrum cephalosporins and carbapenems led to an increase in selective pressure, which favoured the formation of MDR-GNB in G23, carrying multiple resistance genes, such as *Pseudomonas* spp. and PDR *Klebsiella pneumoniae* (as seen in this study), as well as the overdevelopment of *S. maltophilia*. Moreover, it is well known that metallo-beta-lactamases (MBL) producing GNB are resistant to almost all antibiotics, including those recently introduced in therapy (e.g., ceftazidime/avibactam, ceftolozane/tazobactam, imipenem/relebactam), with the exception of the new cefiderocol [40]. Additionally, a continuous high ratio of consumption was recorded for macrolides compared to the span 2011–2019, following the unjustified widespread use of azithromycin and clarithromycin in the treatment of COVID-19 [39]. These could be responsible for the increased representation of the MLSB phenotype noticed in the case of *Staphylococcus aureus* in G23.

In the present study, an important increase in the number of MDR *Pseudomonas* spp. was recorded in 2023 relative to 2020 (*p* = 0.047). As far as G23 is concerned, 27.59% of the strains of these species were XDR, and 37.93% were CR-GNB. All *A. baumannii* strains recorded in 2023 were MDR and over 3/4 were XDR, with carbapenem resistance being reported in 88.24% of the strains. These data are particularly significant if considering the CARMIAAM report [39] which indicated that, in Romania of 2021, *A. baumannii* was 93% resistant to carbapenems, with an extended resistance of 89.6%, placing the country in fourth position among the EARS-Net countries, for both indicators. These figures also explain the identification of *A. baumannii* among the independent predictive variables for negative evolution in our study.

As concerns the *Enterobacteriaceae* group, the greatest impact was exerted by the increased resistance of *Klebsiella* spp. strains. *K. pneumoniae* was the only species in this study for which strains entirely resistant to all classes of tested antibiotics were isolated. The result is relevant if considering that, in Romania of 2021, the multi-drug resistance of *K. pneumoniae* remained at the high level of 45.1%, the fifth level among EARS-Net states [39].

The data obtained underline that the greatest challenge associated with the COVID-19 pandemic in the case of burn-injured patients has been posed by infections with GNB, whose AMR often does not allow too many therapeutic options. 

The consequences of the pandemic for people and public health are still being felt around the world, and efforts to combat AMR are only now starting to balance out following the reorganization of healthcare professionals to assist the COVID-19 response throughout the European Region. Governments from all over the world were forced to understand that more coordinated actions were required. This cleared the path for a more unified front against upcoming health risks, such as AMR. It seems reasonable that such a unified front will be able to combat the impending threat of AMR with greater force in the future. The seven G7 finance ministers decided on 13 December 2021 to increase readiness against the “silent pandemic” of AMR [19]. The Centers for Disease Control and Prevention (CDC) is also targeting grassroots actions to better prepare the US to combat AMR, integrating the One Health approach into this process [36]. Our results are consistent with the global data on AMR that has arisen as a result of antibiotic treatment during COVID-19. Other studies published by our team in the same hospital, in the post-pandemic period, highlighted the same situation of AMR [41,42].

This study provides valuable information on the infectious pathology in burn-injured patients, particularly if considering that our hospital is the largest university hospital in the western region of Romania, hospitalizing patients affected by severe burns from all over the country. It is worth noting that a new centre for severely burned patients is under construction as part of a healthcare hub. In this context, the share of this pathology will be increasing, an additional reason why documentation is necessary through the information brought by the present study.

The novelty of our work consists in clarifying the way the COVID-19 pandemic has affected the evolving pattern of bacterial infections in a burn unit, as well as offering useful insights that could direct future clinical practice and policymaking in burn care and beyond, by examining changes in the variety of bacterial species, resistance patterns, and the effects of pandemic-related policies on infection management and treatment.

Nonetheless, there are certain study limitations that must also be acknowledged, primarily arising from its design and including the unicentric, cross-sectional nature, as well as the limited period (8 months) for each of the two years considered. Limitations arising from the applied microbiological method lie in the use of different standards for the interpretation of susceptibility to antibiotics (the CLSI standard was used until 2022, with EUCAST superseding it in 2023). Another shortcoming was generated by the lack of additional testing to other reserve antibiotics in the case of strains that proved resistant to all antibiotics included in the VITEK kit, which is necessary to certify the PDR phenotype.

## 4. Materials and Methods

A unicentric, cross-sectional study was carried out to analyse the changes of the infectious pattern in patients hospitalized in the BFU of the SCJUPBT Romania, considering similar periods of two years, the year 2020, when the COVID-19 pandemic started in Romania, and the year 2023, when it ended.

Affiliated with the university, the medical facility is a hospital, which comprises 1174 beds and provides medical care for the western region of Romania. The BFU currently consists of 9 rooms equipped with one bed each (of which 5 beds are for intensive care), 2 rooms with 2 beds each, 2 Operating Rooms, Therapeutic Bathrooms, and a Fast Sterilization Room.

On the BFU, antibiotic therapy is carried out according to the Sanford guidelines, adapted to the antibiotic sensitivity testing (AST) and following interdisciplinary discussions with the infectious disease and clinical microbiology doctors. Infectious screening of patients upon admission is also performed; in the case of initial positive MDR cultures, the screening is repeated every 3 days. At that time, all prosthetic materials are replaced, and the general toilet of the patient is performed, including the dressing of burn wounds and collection of microbiological cultures. There are internal protocols for collecting microbiological samples, and the wound cultures are collected sequentially.

This study compared two groups of patients with at least one positive microbiological diagnosis who were admitted to BFU. The first, called Group 2020 (G20), consisted of 48 patients hospitalized between January and August 2020, while the second, called Group 2023 (G23), was comprised 69 patients, hospitalized between January and August 2023. The following information on patients admitted to BFU was collected from the hospital database: age, gender, length of stay, outcome (death or discharge), total body surface area burn percentage (TBSA), depth of the burn [43], ABSI score [44], mechanism of burn. The fatality rate was calculated based on ABSI score. The percentages thus obtained are indicated in Table 7 below. 

A number of 253 clinical samples collected from G23 and 225 clinical samples collected from G20 were studied, with a total of 478 samples for the entire study. The main samples in both groups of patients (G23/G20) were wound secretions (60.07%/56.44%), blood cultures (20.56%/24.88%), bronchial aspirates (10.28%/13.34%), and urine cultures (8.3%/3.56%).

Based on the data collected from G23 and G20, this study comparatively examined the demographic profile of the patients with infections admitted to BFU and highlighted the differences in the etiological spectrum and AMR of the microorganisms isolated from the clinical samples collected from those patients in 2020 and 2023.

The inclusion criteria applicable to G23/G20 were as follows: burn-injured patients over the age of 18, admitted to BFU-SCJUPBT through the Emergency Service, upon request or transferred from other hospitals, and who had at least one microbiological diagnosis of bacterial/fungal infection during hospitalization.

The exclusion criteria were patients who died within the first 24 h of hospitalization, patients with a negative microbiological result, and patients with incomplete data for this study.

### 4.1. Microbiology Procedures

The identification of pathogens, the AST, and the determination of the minimum inhibitory concentration (MIC) were performed according to the protocols of the Microbiology Laboratory, via the VITEK^®^ 2 Compact (BioMerieux, Marcy l’Etoile, France) and Matrix Assisted Laser Desorption/Ionization Time-of-Flight Mass Spectrometry systems (MALDI Biotyper, Bruker, Germany). The interpretation of the AST was carried out pursuant to the corresponding standards (EUCAST in 2023 [46] and CLSI in 2020 [47]).

A positive result repeatedly recorded for the same sample was considered only if the microbial agent was different, or if the same microbial agent presented a different AMR profile.

For clinically important bacteria, the classifications were used according to the acquired phenotypes: Methicillin-resistant *S. aureus* (MRSA): *S. aureus* with MIC ≥ 4 to oxacillin; Extended-spectrum beta-lactamase (ESBL) secreting GNB: resistance to all penicillins/cephalosporins [46,47]; Carbapenem-resistant GNB (CR-GNB): enterobacteria with MIC ≥ 4 to imipenem, meropenem, and non-fermentative GNB with MIC ≥ 8 to imipenem, meropenem; Multi-drug-resistant (MDR) bacteria: resistance to at least one antibiotic from three or more classes of antibiotics active for a given species [48]; Extensively drug-resistant bacteria (XDR): resistance to at least one agent from all antimicrobial classes except one or two classes [48]; Pan-drug-resistant (PDR) bacteria: nonsusceptibility to all agents in all active antimicrobial categories for a particular species [48].

PDR was perceived in this study as widespread resistance to all antibiotics routinely tested in the hospital laboratory, i.e., penicillins, cephalosporins, carbapenems, monobactams, quinolones, aminoglycosides, and colistin. No exhaustive testing was carried out on antibiotics that are not administered to hospitalized patients.

This study was approved by the Ethics Committee of SCJUPBT (414/27 October 2023). The requirements of EU Regulation no. 679/2016 on the processing of personal data were entirely met for the data collected in this study.

### 4.2. Statistical Method

The databases were analysed via the IBM SPSS Statistics 20 program (SPSS Inc., Chicago, IL, USA). Numerical variables were conveyed by median and interquartile range (IQR), while nominal variables were conveyed by value and percentage. Data distribution testing was performed via the Shapiro–Wilk test.

Numerical variables were compared via the Mann–Whitney U non-parametric statistical test for independent samples. Nominal variables were compared by the hi^2^ test (Fisher’s Exact Test). For correlation purposes, the Spearman’s Rank Correlation Coefficient was applied. Variables with statistically significant changes (*p* ≤ 0.05) and variance inflation factor (VIF) below 3 (calculated by linear regression to avoid multicollinearity) were included in the logistic regression model. The model was chosen considering the Nagelkerke R^2^ Coefficient and the test for deviation measurement, grounded in the theoretical model suggested by statisticians Hosmer and Lemeshow. The threshold value for statistical significance was considered ≤0.05 and two-tailed tests were used.

## 5. Conclusions

This study identified age, burned body surface, mechanical ventilation, haemodialysis, and infection with *Acinetobacter* spp. as independent predictive variables for negative evolution. The analysis of the incidence of identified bacterial species indicated comparable results between the two groups.

The increased incidence of non-fermenting GNB must also be acknowledged, given the threat posed for burn-injured patients. These bacteria can proliferate in the wound exudate, forming biofilms and producing infections in critically ill and immunosuppressed patients. The AMR increase marked in 2023 was apparent only in the strains of *Pseudomonas* spp. and *Klebsiella* spp., the latter being often resistant to the entire panel of antibiotics tested.

These findings call for the careful monitoring of resistance trends in isolates identified in the BFU, as well as in other hospital wards. Concerted efforts aimed at enhancing the appropriate use of antibiotics, reinforcing the healthcare associated infections (HAI) prevention and control measures, and supporting the continuous training program for the medical and auxiliary staff, are still essential to diminish the impact of bacterial resistance in the future.

## Figures and Tables

**Table 1:** The demographic and prognostic data of the patients on admission.

Variable	2020		2023		
	Nr. (%)	95%CI *	Nr. (%)	95%CI	*p*
Patients	N1 = 48		N2 = 69		
Women patients, F (%)	16 (33.33)	[20.40–48.41%]	20 (28.99)	[18.69–41.16%]	0.685
Male patients, M (%)	32 (66.67)	[51.59–79.60%]	49 (71.01)	[58.84–81.31%]	
Median Age [IQR]	59.00 [47.50–70.50]	/	49.00 [35.00–61.00]	/	0.005
Patients aged 18–19 (%)	1 (2.08)	[0.05–11.07%]	1 (1.45)	[0.04–7.81%]	1.00
Patients aged 20–29 (%)	0 (0)	/	10 (14.49)	[7.17–25.04%]	0.005
Patients aged 30–39 (%)	5 (10.42)	[3.47–22.66%]	12 (17.39)	[9.32–28.41%]	0.424
Patients aged 40–49 (%)	8 (16.67)	[7.48–30.22%]	12 (17.39)	[9.32–28.41%]	1.00
Patients aged 50–59 (%)	10 (20.83)	[10.47–34.99%]	15 (21.74)	[12.71–33.31%]	1.00
Patients aged 60–69 (%)	11 (22.92)	[12.03–37.31%]	9 (13.04)	[6.14–23.32%]	0.212
Patients aged 70–79 (%)	5 (10.42)	[3.47–22.66%]	7 (10.14)	[4.18–19.79%]	1.00
Patients aged 80–89 (%)	7 (14.58)	[6.07–27.76%]	2 (2.90)	[0.35–10.08%]	0.031
Patients aged 90–99 (%)	1 (2.08)	[0.05–11.07%]	1 (1.45)	[0.04–7.81%]	1.00
Median length of stay [IQR]	20.00 [12.00–52.00]	/	19 [8.00–41.00]	/	0.745
Median fatality [IQR]	3.00 [2.00–4.00]	/	2.00 [1.00–3.00]	/	0.023

* 95%CI: 95% confidence interval.

**Table 2:** Distribution by fatality categories.

Fatality Rate	0–1%	2%	10–20%	30–50%	60–80%	>80%
G23 nr. (%)	5 (7.25)	17 (24.64)	16 (23.19)	16 (23.19)	6 (8.69)	9 (13.04)
G20 nr. (%)	0	7 (14.58)	8 (16.67)	15 (31.25)	11 (22.92)	7 (14.58)

**Table 3:** Independent predictive variables for negative evolution.

Variable	Fatal Outcome		Positive Evolution					
	N1 = 33	95%CI *	N2 = 80	95%CI	*p*	OR	*p*	HR
Median age [IQR]	64.00 [49.00–71.00]	/	50.00 [35.00–62.00]	/	0.005	1.04 [1.01–1.06]	0.002	1.12 [1.04–1.21]
Body surface [IQR]	40.00 [16.00–65.00]	/	14.50 [5.50–24.50]	/	<0.001	1.06 [1.03–1.08]	0.001	1.12 [1.05–1.21]
Burn dressing ≥10% of BS (%)	23 (69.70)	[51.29–84.41%]	38 (47.50)	[36.21–58.98%]	0.039	2.54 [1.07–6.02]	0.008	0.02 [0.00–0.37]
Ventilatory support (%)	27 (81.82)	[64.54–93.02%]	13 (16.25)	[8.95–26.18%]	<0.001	23.19 [7.99–67.31]	0.002	37.32 [3.65–381.05]
Haemo-dialysis (%)	13 (39.39)	[22.91–57.86%]	2 (2.50)	[0.30–8.74%]	<0.001	25.35 [5.29–121.57]	0.006	47.11 [2.99–740.36]
Patients infected with								
*Acinetobacter* spp. (%)	21 (63.64)		19 (23.75)		<0.001	5.61 [2.34–13.49]	0.019	9.02 [1.44–56.62]

* 95%CI: 95% confidence interval.

**Table 4:** Distribution of the main bacterial genera/species isolated in clinical samples (2020 vs. 2023).

Species	2020		2023		
	Nr. (%)	95%CI *	Nr. (%)	95%CI	*p*
Wound secretions					
*S. aureus*	23 (16.31)	[10.63–23.46%]	31 (18.45)	[12.90–25.16%]	0.654
*Enterococcus* spp.	21 (14.89)	[9.46–21.86%]	22 (13.10)	[8.39–19.15]	0.366
*Pseudomonas* spp.	19 (13.48)	[8.31–20.24%]	21 (12.50)	[7.91–18.47%]	0.865
*Bacillus cereus*	5 (3.55)	[1.16–8.08%]	20 (11.90)	[7.43–17.79%]	0.010
*Acinetobacter* spp.	19 (13.48)	[8.31–20.24%]	19 (11.31)	[6.95–17.10%]	0.604
*Klebsiella* spp.	13 (9.22%)	[5.00–15.25%]	16 (9.52)	[5.54–15.01%]	1.00
CNS	10 (7.09)	[3.45–12.66%]	1 (0.60)	[0.02–3.27%]	0.032
Blood cultures					
CNS	21 (36.21)	[23.99–49.88%]	15 (28.85)	[17.13–43.08%]	0.424
*S. aureus*	7 (12.07)	[4.99–23.30%]	12 (23.08)	[12.53–36.84%]	0.139
*A. baumannii*	12 (20.69)	[11.17–33.35%]	8 (15.38)	[6.88–28.08%]	0.621
*Klebsiella* spp.	2 (3.45)	[0.42–11.91%]	3 (5.77)	[1.21–15.95%]	0.665
*Enterococcus* spp.	2 (3.45)	[0.42–11.91%]	3 (5.77)	[1.21–15.95%]	0.665
Bronchial aspirates					
*A. baumannii*	12 (35.29)	[19.75–53.51%]	7 (23.33)	[9.93–42.28%]	0.412
*S. maltophilia* ^1^	0 (0)	/	4 (13.33)	[3.76–30.72%]	0.043
*Pseudomonas* spp.	4 (11.76)	[3.30–27.45%]	3 (10.00)	[2.11–26.53%]	1.00
*S. Paucimobilis* ^2^	0 (0)	/	2 (6.67)	[0.82–22.07%]	0.216
*Klebsiella* spp.	2 (5.88)	[0.72–19.68%]	2 (6.67)	[0.82–22.07%]	1.00

^1^ *Stenotrophomonas maltophilia*, ^2^ *Sphingomonas paucimobilis*, * 95%CI: 95% confidence interval.

**Table 5:** Comparative distribution by categories of pathogens (2020 vs. 2023).

Strains	2020		2023		
	N = 245 (%)	95%CI *	N = 245 (%)	95%CI	*p*
GNB of which:	134 (54.69)	[48.23–61.04%]	140 (51.28)	[45.18–57.35%]	0.480
Non-fermenting	74 (30.20)	[24.52–36.37%]	76 (27.84)	[22.61–33.56%]	0.562
GPC	101 (41.22)	[35.00–47.67%]	123 (45.05)	[39.05–51.17%]	0.424
Fungi	10 (4.08)	[1.97–7.38%]	10 (3.66)	[1.77–6.63%]	0.823

* 95%CI: 95% confidence interval.

**Table 6:** Comparative distribution of resistance phenotypes in clinical isolates (2020 vs. 2023).

	2020		2023		
PHENOTYPE	Nr. (%)	95%CI *	Nr. (%)	95%CI	*p*
Species: *Acinetobacter baumannii* TOTAL = 46/34					
**MDR**	45 (97.83)	[88.47–99.94%]	34 (100)	[89.72–100.00%]	1.00
**XDR**	28 (60.87)	[45.37–74.91%]	26 (76.47)	[58.83–89.25%]	0.156
**PDR**	0 (0)	/	0 (0)	/	/
**CR-GNB**	42 (91.30)	[79.21–97.58%]	30 (88.24)	[72.55–96.70%]	0.717
**R-SXT**	40 (86.96)	[73.74–95.06%]	30 (88.24)	[72.55–96.70%]	1.00
Species: *Staphylococcus aureus* TOTAL = 34/49					
**MRSA**	14 (41.18)	[24.65–59.30%]	22 (44.90)	[30.67–59.77%]	0.823
**MDR**	20 (58.82)	[40.70–75.35%]	25 (51.02)	[36.34–65.58%]	0.510
**MLSB**	10 (29.41)	[15.10–47.48%]	22 (44.90)	[30.67–59.77%]	0.176
**R-FQ**	7 (20.59)	[8.70–37.90%]	16 (32.65)	[19.95–47.54%]	0.319
**R-SXT**	8 (23.53)	[10.75–41.17%]	4 (8.16)	[2.27–19.60%]	0.062
Species: Coagulase-negative staphylococci TOTAL = 33/16					
**MRCNS**	11 (33.33)	[17.96–51.83%]	10 (62.50)	[35.43–84.80%]	0.069
**MLSB**	0 (0)	/	2 (12.50)	[1.55–38.35%]	0.102
Species: *Pseudomonas* spp. TOTAL = 27/29					
**MDR**	5 (18.52)	[6.30–38.08%]	13 (44.83)	[26.45–64.31%]	0.047
**CR-GNB**	7 (25.93)	[11.11–46.28%]	11 (37.93)	[20.69–57.74%]	0.399
**XDR**	4 (14.81)	[4.19–33.73%]	8 (27.59)	[12.73–47.24%]	0.334
**PDR**	0 (0)	/	0 (0)	/	/
Species: *Enterococcus* spp. TOTAL = 23/25					
**VRE**	1 (4.35)	[0.11–21.95%]	1 (4.00)	[0.10–20.35%]	1.00
Species: *Klebsiella* spp. TOTAL = 18/22					
**MDR**	2 (11.11)	[1.38–34.71%]	5 (22.73)	[7.82–45.37%]	0.427
**XDR**	3 (16.67)	[3.58–41.42%]	1 (4.55)	[0.12–22.84%]	0.333
**PDR**	1 (5.55)	[0.14–27.29%]	9 (40.91)	[20.71–63.65%]	0.013
**ESBL**	8 (44.44)	[21.53–69.24%]	11 (50)	[28.22–71.78%]	0.761
**CR-GNB**	6 (33.33)	[13.34–59.01%]	12 (54.55)	[32.21–75.61%]	0.216
**R-AG**	4 (22.22)	[6.41–47.64%]	11 (50)	[28.22–71.78%]	0.104
**R-FQ**	5 (27.78)	[9.69–53.48%]	13 (59.09)	[36.35–79.29%]	0.062
**R-SXT**	3 (16.67)	[3.58–41.42%]	11 (50)	[28.22–71.78%]	0.045

* 95%CI: 95% confidence interval. **Legend:** MDR: multi-drug-resistant, XDR—extensive drug resistance, PDR—pan-drug resistance, CR-GNB—Carbapenem-resistant Gram-Negative Bacteria, ESBL—Extended spectrum beta-lactamase, MRSA—methicillin-resistant *S. aureus*, MRCNS—methicillin-resistant Coagulase negative staphylococci, VRE—vancomycin-resistant *Enterococcus*, MLSB: macrolide-lincosamide-streptogramin B resistance, R-AG—resistance to aminoglycosides (AG), R-FQ resistance to fluoroquinolones (FQ), R-SXT—resistance to Trimethoprim/sulfamethoxazole (SXT).

**Table 7:** Fatality rate calculation (Usmani, amended) [45].

ABSI	Life Threat	Survival Probability (%)	Fatality Rate (%)
2–3	Very low	≥99%	≤1%
4–5	Moderate	98%	2%
6–7	Moderately severe	80–90%	10–20%
8–9	Serious	50–70%	30–50%
10–11	Severe	20–40%	60–80%
≥12	Maximal	≤10%	≥90%

## Data Availability

Data are contained within the article.
